# Intramuscular progesterone versus 8% Crinone vaginal gel for luteal phase support following blastocyst cryopreserved single embryo transfer: a retrospective cohort study

**DOI:** 10.1186/s40738-020-00079-y

**Published:** 2020-07-01

**Authors:** Jennifer B. Bakkensen, Catherine Racowsky, Ann M. Thomas, Andrea Lanes, Mark D. Hornstein

**Affiliations:** Department of Obstetrics & Gynecology, Brigham & Women’s Hospital and Harvard Medical School, 75 Francis Street, Boston, MA 02115 USA

**Keywords:** Luteal support, Intramuscular progesterone, Vaginal progesterone, Crinone, IVF, Cryo embryo transfer

## Abstract

**Background:**

The optimal route of progesterone administration for luteal support in cryopreserved embryo transfer (CET) has been the subject of much debate. While most published research has pertained to day 3 transfers, recent data on blastocyst CET has suggested that intramuscular progesterone (IMP) is superior to twice daily vaginal Endometrin suppositories for luteal phase support, resulting in significantly higher ongoing pregnancy rates. This study aimed to determine whether IMP is similarly superior to 8% Crinone vaginal gel for luteal phase support following blastocyst CET.

**Methods:**

Autologous and donor oocyte blastocyst cryopreserved single embryo transfer (SET) cycles from January 2014–January 2019 utilizing either 50 mg IMP daily or 90 mg 8% Crinone gel twice daily for luteal support were included. The primary outcome was live birth. Secondary outcomes included biochemical pregnancy, spontaneous abortion, and clinical pregnancy. All analyses were adjusted a priori for oocyte age. Log-binomial regression analysis was performed with differences in outcomes reported as relative risk (RR) with 95% confidence intervals (CI).

**Results:**

A total of 1710 cycles were included, of which 1594 utilized IMP and 116 utilized 8% Crinone gel. Demographic and cycles characteristics were similar between the two groups. Compared to cycles utilizing IMP, cycles utilizing Crinone gel resulted in similar rates of live birth (RR 0.91; 95% CI 0.73–1.13), biochemical pregnancy (RR 1.12, 95% CI 0.65–1.92), spontaneous abortion (RR 1.41, 95% CI 0.90–2.20), and clinical pregnancy (RR 1.00, 95% CI 0.86–1.17).

**Conclusions:**

Compared to cryopreserved blastocyst SET cycles utilizing IMP for luteal support, cycles utilizing 8% Crinone gel resulted in similar likelihood of live birth.

## Background

Progesterone is required for the establishment and maintenance of early pregnancy until the luteo-placental shift, which typically occurs at about 7–10 weeks’ gestation [1–3]. It is necessary to provide exogenous progesterone to women undergoing both fresh embryo transfer, in which endogenous progesterone is decreased due to gonadotropin-releasing hormone (GnRH) agonist and antagonist ovulation induction protocols and aspiration of granulosa cells at oocyte retrieval [4–7], and programmed cryopreserved embryo transfer (CET), in which endogenous progesterone production is minimal [8].

The optimal route of progesterone administration has been the subject of much debate and ongoing research, with no single formulation or regimen identified as superior [9]. Oral formulations are commonly avoided as they have been shown to have poor bioavailability and are associated systemic adverse events such as drowsiness, dizziness, and headaches [10–12]. A strong body of evidence from fresh embryo transfer cycles has supported the equivalence of vaginal progesterone as compared to intramuscular progesterone (IMP) in terms of pregnancy and live birth outcomes [9, 13–20]. Data from CET cycles, however, are less conclusive.

A 2010 Cochrane review found no difference in pregnancy rates between the use of IMP or vaginal progesterone for luteal phase support [21]; however, only one of the four trials included in that analysis featured CET, and the embryos in that trial were cleavage stage embryos [22], rendering these results less applicable to current practice. Retrospective studies limited to CET have also largely focused on cleavage stage embryo transfers and have yielded similarly conflicting results [23–25], with a more recent meta-analysis concluding insufficient evidence to recommend any one protocol [26].

The strongest data to date in comparing IMP vs. vaginal progesterone among blastocyst stage CET derive from a 2018 randomized controlled trial published by Devine et al. which found that blastocyst CET cycles supported by twice daily vaginal Endometrin suppositories resulted in significantly lower ongoing pregnancy rates than cycles supported with either IMP alone or a combination of IMP plus Endometrin [27]. However, the generalizability of these data remains in question, as the inferior clinical outcomes obtained using twice daily vaginal Endometrin may not necessarily apply to all vaginal progesterone preparations and dosing regimens.

The goal of the present study is to assess the efficacy of IMP versus 8% Crinone vaginal gel for luteal support in blastocyst CET by comparing pregnancy and live birth outcomes between the two groups.

## Methods

### Study design

All autologous and donor egg vitrified day 5 or day 6 single embryo transfer (SET) cycles at the Brigham and Women’s Hospital from January 1, 2014, to January 15, 2019 were retrospectively reviewed for the type of progesterone used for luteal phase support. Patient demographics, embryo and cycle characteristics, and clinical outcomes were compared between those supported with standard dose IMP in oil (locally compounded at Village Fertility, Waltham, Massachusetts, or Freedom Fertility, Byfield, Massachusetts) and those supported with vaginal progesterone gel (8% Crinone, Actavis Pharma, Inc). Exclusion criteria included cryopreservation stage other than blastocyst, non-standard dosages of IMP or Crinone, alternate formulations of progesterone, and cycles not requiring luteal support (i.e., natural cycles).

Data were collected from a prospectively maintained departmental database and the hospital electronic medical record system.

### Clinical and laboratory protocols

Controlled ovarian hyperstimulation was performed utilizing GnRH antagonists, GnRH agonists, estradiol priming, or low-dose GnRH agonist flare protocols [28–31]. Following retrieval, oocytes were either inseminated in groups (3–5 oocytes) or underwent intracytoplasmic sperm injection (ICSI) using routine procedures. A fertilization check was performed at 16–18 h and zygotes with 2 pronuclei were cultured individually in 25 uL drops of Global Total medium (Life Global Group, Cooper Surgical; Guildford, CT) overlain with mineral oil in *Esco Miri*® Benchtop Multi-room Incubators. On day 3, embryos were moved to fresh drops of Global Total medium and on day 5 were either cryopreserved or cultured for a further 24 h for re-evaluation of cryopreservation suitability on day 6. A modification of the Gardner grading system was used to grade the quality of all embryos, with embryo quality classified as good, fair, or poor according to the Society for Assisted Reproductive Technology (SART) embryo grading system [32]. All embryos were evaluated and scored on day 5 and, if culture continued to day 6, again on day 6. If pre-implantation genetic testing (PGT) was indicated, biopsy was performed at the blastocyst stage prior to cryopreservation. Indications for PGT included aneuploidy screening (PGT-A), testing for monogenic disorders (PGT-M), and testing for chromosomal structural rearrangements (PGT-SR). Blastocysts were cryopreserved individually using the Cryolock vitrification device (Irvine Scientific, Santa Ana, CA) and warmed using the manufacturer recommended warming protocol.

Uterine preparation was carried out using oral, vaginal, or transdermal 17-beta estradiol with the aim of achieving an endometrial thickness of 7 mm by ultrasound. The day of embryo transfer was determined according to internal protocols and patient history. For cycles utilizing IMP for luteal support, IMP was initiated at a dose of 25 mg starting the evening 5 days prior to transfer followed by 50 mg daily. For cycles utilizing 8% Crinone vaginal gel, one applicator (90 mg) of Crinone was prescribed twice daily. Eleven days after embryo transfer, patients had serum human chorionic gonadotropin (hCG) levels checked at 2-day intervals. If the serial hCG rise was appropriate, an obstetric ultrasound was performed at 7–8 weeks of gestational age. If clinical pregnancy was confirmed, luteal support was continued through 10 weeks of gestation.

### Clinical outcomes

Our primary outcome was live birth, defined as at least one liveborn infant. Secondary outcomes included biochemical pregnancy (hCG > 3 IU/L) with no gestational sac identified on ultrasound), spontaneous abortion (loss of clinical pregnancy before 20 weeks’ gestation), and clinical pregnancy (gestational sac documented by ultrasonography at 7–8 weeks’ gestation).

### Statistical analysis

Log binomial regression was used to estimate the relative risk (RR) and 95% CI for all clinical outcomes. All analyses controlled a priori for oocyte age at time of cryopreservation. Generalized estimating equations were used to account for correlations between multiple cycles from the same woman. Additional variables tested as potential confounders included carrier BMI, endometrial thickness, embryo quality, and freeze day; however, these variables did not confound the exposure estimate by > 10% and were therefore not included in the final model [33]. Statistical analyses were performed using SAS version 9.4 software (Cary, NC, USA) [34].

## Results

A total of 1594 IMP and 116 8% Crinone SET cycles were included. Demographic and cycle characteristics were similar between the two groups as outlined in Tables 1 and 2, respectively. Among cycles supported with IMP, 310 (19.5%) embryos were biopsied prior to transfer, including 169 PGT-A (10.6%), 67 PGT-M (4.2%), 46 PGT-A and PGT-M (2.9%), 13 PGT-SR (0.82%), and 15 PGT-SR and PGT-A (0.94%). Among cycles supported with 8% Crinone, 18 (15.5%) embryos were biopsied prior to transfer, including 9 PGT-A (7.8%), 6 PGT-M (5.2%), and 3 PGT-A and PGT-M (2.6%).

**Table 1:** Patient demographics for blastocyst cryopreserved SET cycles supported with IMP versus 8% Crinone vaginal gel

	IMP (***n*** = 1594)	Crinone (***n*** = 116)
Oocyte age at cryopreservation (y)	33.9 ± 4.1	34.2 ± 3.9
Age of recipient at transfer (y)	35.6 ± 4.2	35.6 ± 4.0
Gravidity		
0	671 (42.1)	52 (44.8)
≥ 1	923 (57.9)	64 (55.2)
Parity		
0	1001 (62.8)	74 (63.8)
≥ 1	593 (37.2)	42 (36.2)
Prior SAB		
Yes	1137 (71.3)	88 (75.9)
No	457 (28.7)	28 (24.1)
BMI of embryo recipient (kg/m^2^)	25.3 ± 6.0	25.4 ± 5.7
No. obese recipients ^a^	257 (16.1)	12 (10.3)
Primary infertility diagnosis		
Unexplained	446 (28.0)	46 (39.7)
Male factor	385 (24.2)	25 (21.6)
Tubal factor	85 (5.3)	5 (4.3)
Anovulation	195 (12.2)	13 (11.2)
Endometriosis	72 (4.5)	6 (5.2)
Diminished ovarian reserve	168 (10.5)	4 (3.5)
Uterine factor^b^	34 (2.1)	0 (0.0)
Gestational carrier	19 (1.2)	0 (0.0)
Other	190 (11.9)	17 (14.7)

Values represent n (%) or mean ± SD. *SET* single embryo transfer, *IMP* intramuscular progesterone, *SAB* spontaneous abortion, *BMI* body mass index

^a^ Obesity defined as BMI ≥ 30 kg/m^2^

^b^ Uterine factors include adenomyosis, exposure to DES, fibroids, intrauterine synechiae, or a unicornuate system

**Table 2:** Embryo and cycle characteristics for blastocyst cryopreserved SET cycles supported with IMP versus 8% Crinone vaginal gel

	IMP (***n*** = 1594)	Crinone (***n*** = 116)
Autologous vs. donor embryos		
Autologous	1502 (94.2)	113 (97.4)
Donor	92 (5.4)	3 (2.6)
ICSI	837 (52.2)	60 (51.8)
Day of embryo cryopreservation		
Day 5	1333 (83.6)	94 (81.0)
Day 6	261 (16.4)	22 (19.0)
Endometrial thickness at trigger/mapping (mm)	9.5 ± 2.7	9.6 ± 2.6
Biopsied embryos	310 (19.5)	18 (15.5)
Day of embryo transfer		
Day 5	1556 (97.6)	115 (99.1)
Day 6	38 (2.4)	1 (0.9)
Embryo quality ^a, b^		
Good	652 (40.9)	51 (44.7)
Fair	444 (27.9)	31 (27.2)
Poor	497 (31.2)	32 (28.1)

Values represent n (%) or mean ± SD. *SET* single embryo transfer, *IMP* intramuscular progesterone, *ICSI* intracytoplasmic sperm injection

^a^ Embryo quality data missing for 1 IMP cycle and 2 Crinone cycles

^b^ Embryo quality defined according to the SART grading system

Clinical outcomes are outlined in Table 3. Of cycles supported with IMP, 47.4% achieved live birth, versus 41.4% of those supported with 8% Crinone. However, this difference did not achieve statistical significance (RR 0.91, 95% CI 0.73–1.13). Similarly, there was no significant difference between those receiving IMP versus those receiving Crinone gel in the rates of biochemical pregnancy (RR 1.12, 95% CI 0.65–1.92), spontaneous abortion (RR 1.41, 95% CI 0.90–2.20), or clinical pregnancy (1.00, 95% CI 0.86–1.17).

**Table 3:** Clinical outcomes for blastocyst cryopreserved SET cycles supported with IMP versus 8% Crinone vaginal gel

Clinical outcome^a^	IMP (***n*** = 1594)	Crinone (***n*** = 116)	RR (95% CI)^b^
Live birth^c^	755 (47.4)	48 (41.4)	0.91 (0.73, 1.13)
Biochemical pregnancy	164 (10.3)	13 (11.2)	1.12 (0.65, 1.92)
Spontaneous abortion	163 (10.2)	17 (14.7)	1.41 (0.90, 2.20)
Clinical pregnancy	949 (59.5)	68 (58.6)	1.00 (0.86, 1.17)

Values represent n (%). *SET* single embryo transfer, *IMP* intramuscular progesterone, *RR* Relative risk, *CI* confidence interval

^a^All values expressed as percentage of all transfers

^b^Models adjusted a priori for patient age at embryo cryopreservation. Generalized estimating equations were used to account for correlations between multiple cycles from the same woman

^c^Three patients having received IMP were lost to follow-up after confirmation of clinical pregnancy and were therefore excluded from the live birth analysis.å

## Discussion

The goal of this study was to assess the efficacy of IMP versus 8% Crinone gel for luteal support in blastocyst CET. This study found that rates of live birth, biochemical pregnancy, spontaneous abortion, and clinical pregnancy were similar between the two groups.

These findings are in contrast to those from a recent randomized controlled trial published by Devine et al., which found that IMP was superior to twice daily vaginal progesterone for luteal support. In that study, 645 patients undergoing blastocyst CET were randomized to receive either 50 mg daily IMP, 200 mg twice daily vaginal Endometrin suppositories plus 50 mg IMP every third day, or 200 mg twice daily vaginal Endometrin suppositories alone. An interim analysis found that while the groups initially had similar pregnancy rates, the ongoing pregnancy rate among the Endometrin-only group was significantly lower than in either of the groups receiving IMP (31% vs. 50 and 47% respectively, *p* = 0.004) [27]. While this interim analysis did not include live birth results, it is interesting to note that although the ongoing pregnancy rate in the IMP arm of the Devine study is similar to the live birth rate in the IMP arm of our own study (50% vs. 47%, respectively), the ongoing pregnancy rate of the vaginal progesterone group in Devine study (31%) is far inferior to the live birth rate of the vaginal progesterone group in the present study (41.4%).

While we await the published live birth data from the Devine study, we postulate that the apparent discrepancy between the interim analysis and our own findings may at least in part be attributable to the different vaginal progesterone preparations studied – i.e., Endometrin suppositories versus 8% Crinone gel. A review of the literature specifically comparing IMP to Crinone among blastocyst CET reveals that our results are consistent with those of a study by Shapiro et al., which found that among 920 autologous and donor blastocyst CET, implantation rates, clinical pregnancy rates, and live birth rates were similar between cycles supported with IMP versus those supported with 8% Crinone gel [23]. Together with the results of the present study, these findings suggest that IMP may be equivalent to Crinone in providing luteal support in blastocyst CET.

The varying efficacy of different progesterone preparations may in part be explained by differences in pharmacokinetic properties. It has been suggested that inappropriate levels of progesterone may alter the timing of luteinization, thereby disrupting the window of implantation [35]. In comparing Endometrin vaginal suppositories with Crinone vaginal gel, Endometrin has been shown to produce higher target tissue concentrations, reach steady state more rapidly, and achieve clearance more quickly [36]. Because the optimal timing and dosage of each has yet to be established, it is possible that the standard regimens currently used in CET cycles may yield suboptimal serum or tissue levels, thereby causing dyssynchrony between the endometrium and the embryo and interfering with implantation and early development. Further studies are warranted to investigate these potential mechanisms and establish the optimal regimen for luteal support in CET.

The present study had several notable strengths. It was conducted at a single center with uniformity of clinical and laboratory protocols for the duration of the study period. It included both autologous and donor cycles, enhancing the generalizability of the results. Furthermore, it is one of only two published studies comparing IMP to 8% Crinone gel specifically among blastocyst as opposed to cleavage stage CET, and the only study to our knowledge to be limited to SET, rendering our data highly relevant to current practice.

Our study is limited by its retrospective design, in that women were assigned to receive IMP versus Crinone largely per provider preference as opposed to randomization, which could be a source of unidentified confounding. Furthermore, the number of Crinone cycles included was relatively small. It is thus possible that with a larger sample size, some of the differences in pregnancy and live birth rates may have reached statistical significance. A larger, prospective study is warranted to investigate these findings further.

## Conclusions

The results of this small retrospective study suggest that IMP and 8% Crinone gel may be equivalent in providing luteal support among blastocyst cryopreserved SET as indicated by clinical pregnancy and live birth rates. These findings contribute to a broader understanding of how different formulations of progesterone may support implantation and pregnancy.

## Data Availability

The datasets used and/or analyzed during the current study are available from the corresponding author on reasonable request.

## Abbreviations

ART: Assisted reproductive technology
BMI: Body mass index
CET: Cryopreserved embryo transfer
CI: Confidence interval
GnRH: Gonadotropin releasing hormone
ICSI: Intracytoplasmic sperm injection
IMP: Intramuscular progesterone
hCG: human chorionic gonadotropin
PGT-A: Pre-implantation genetic testing aneuploidy
PGT-M: Pre-implantation genetic testing monogenic
PGT-SR: Pre-implantation genetic testing structural rearrangement
RR: Relative risk
SAB: Spontaneous abortion
SART: Society for Assisted Reproductive Technology
SET: Single embryo transfer
